# False Memories of Familiar Faces: The Role of Familiarity in Recognition and Source Memory

**DOI:** 10.1027/1618-3169/a000631

**Published:** 2025-01-09

**Authors:** Daniella K. Cash, Megan H. Papesh, Alan T. Harrison

**Affiliations:** ^1^ Department of Psychology and Philosophy, Sam Houston State University, Huntsville, TX, USA; ^2^ Department of Psychology, University of Massachusetts - Lowell, Lowell, MA, USA; ^3^ Department of Psychology, Louisiana State University, Baton Rouge, LA, USA

**Keywords:** recognition, source memory, familiarity, facial recognition, associative memory

## Abstract

**Abstract:** Prior familiarity has been shown to increase memory for faces, but different effects emerge depending on whether the face is experimentally or pre-experimentally familiar to the observer. Across two experiments, we compared the effect of experimental and pre-experimental familiarity on recognition and source memory. Pre-experimentally familiar faces were nameable US celebrities, and unfamiliar faces were unnamable European celebrities. Within both sets, faces could be made experimentally familiar via repetition during the learning phase (studied once or thrice). At test, all studied identities were represented by novel (i.e., not studied) photos, allowing us to test memory for the identity rather than the picture. In Experiment 1, repeated presentations of both face types increased recognition rates, but accuracy was generally higher for pre-experimentally familiar faces. Experiment 2 expanded on these findings by pairing the faces with background locations and manipulating associative strength of the face-location pairs. Although pre-experimentally familiar faces were again recognized more often, they were also more likely to be falsely labeled as “old” when paired with new background locations. These results have implications for basic and applied studies examining familiar versus unfamiliar face recognition.


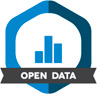


Recognizing familiar faces is typically relatively easy (see Burton, 2013), but many social and professional situations require that people not only recognize a face but know the context in which that person has been encountered previously. For example, as in Mandler’s (1980) famous “butcher on the bus” example, you might come across someone you feel is familiar (your supermarket’s butcher) but fail to retrieve the episodic context that underlies that familiarity (e.g., their name or how you know them). Retrieving information associated with someone’s face, such as from where you know them (i.e., source memory; Johnson et al., 1993), ensures smooth social interactions in real-world face recognition (Johnson & Raye, 1981). Research has shown that there are many factors that influence the likelihood that someone will correctly recognize faces, including the frequency of exposure, depth of processing, and the availability of additional social conceptual information (Devue et al., 2019; Read, 1995; Schwartz & Yovel, 2019). An obvious, but less often studied, factor is the familiarity of the to-be-remembered person. Despite the ubiquity of engaging with people of varying degrees of familiarity daily, little research has explored how variations in familiarity influence recognition and source memory for faces.

Unlike recognition memory for unfamiliar faces, prior work has shown that people are quite accurate at recognizing familiar faces (e.g., Bird et al., 2011; Klatzky & Forrest, 1984; Meltzer, 2023). These accuracy differences are likely due to the differential processing and memorial benefits that are afforded to more extensive mental representations for familiar faces (see Burton, 2013; Johnston & Edmonds, 2009, for reviews). There are multiple ways that researchers have studied the effects of familiarity on face perception and recognition in the lab. One approach is to use faces that are familiar to participants prior to the study. Paradigms that employ pre-experimentally familiar stimuli often use photographs of celebrities or people associated with the participants outside of the study as to-be-remembered stimuli (e.g., Bird et al., 2011; Dobbins & Kroll, 2005; Ellis et al., 1979; Jenkins et al., 2011). Researchers can also experimentally induce familiarity, for example, by showing participants unfamiliar faces multiple times throughout the experiment (e.g., Tulving & Schacter, 1990). Relative to faces made familiar through intra-experimental repetition, pre-experimentally familiar faces are more likely to be recognized, particularly when characteristics like expression or pose change across study and test images (Bird et al., 2011; Bruce, 1982; Dobbins & Kroll, 2005; Ellis et al., 1979; Klatzky & Forrest, 1984; Schwaninger et al., 2002). This difference may be due to more or potentially richer cues (e.g., conceptual information) that are associated with pre-experimentally familiar faces, factors that are difficult to replicate through mere laboratory exposure (Akan & Benjamin, 2023; Patterson & Baddeley, 1977; Schwartz & Yovel, 2019).

As summarized by Akan and Benjamin (2023), several aspects of the extant literature on the effects of face familiarity may limit generalizability. For example, researchers often treat familiarity as a binary construct, overlooking instances where there might be weaker or graded degrees of familiarity (see also Vallano, Slapinski et al., 2019). Likely owing to the challenge of aggregating pre-experimentally familiar faces, many studies examining familiarity do so with small sample sizes, and some also include manipulations of other independent variables. There may also exist confounds across famous and nonfamous faces used in experiments on familiarity: For example, celebrities are often attractive or otherwise distinctive, which is part of why they are famous to begin with, and known individuals are often associated with conceptual, rather than purely perceptual, information. While familiarity clearly influences face recognition, manipulations that better capture real-world nuances (e.g., gradations of familiarity) would improve understanding. Moreover, although face recognition is an important component of personal knowledge about others, recognizing a person is often a more complex retrieval task.

In daily life, it is often not enough to merely know that one has seen a face; people must also remember the context in which the person was encountered. For example, someone might seem familiar, but you may not know if this is because they are a student in your class, your local barista, or someone from a completely different context. This example illustrates a source memory issue. Source memory involves not only recognizing a face or item but also remembering it with important associated features such as time, place, thoughts, etc. (Johnson et al., 1993). Thus, accurately remembering a person can be conceptualized as a source memory task: People must remember both the face and from where that face is known.

As with face recognition, familiarity has been shown to influence source memory accuracy (Kim et al., 2012; Lee et al., 2020; Poppenk, Köhler, & Moscovitch, 2010; Poppenk, McIntosh, et al., 2010; Poppenk & Norman, 2012), with effects that differ for stimuli that are pre-experimentally familiar or familiar through experimental procedures. Studies that used pre-experimentally unfamiliar stimuli found that experimentally inducing familiarity (via repetition) improved source memory accuracy (Poppenk, Köhler, & Moscovitch, 2010; Poppenk, McIntosh, et al., 2010; Poppenk & Norman, 2012). In contrast, research using pre-experimentally familiar items found that increased repetition *decreased* source memory accuracy (Kim et al., 2012). These equivocal findings were later reconciled by Lee et al. (2020), who incorporated both experimentally and pre-experimentally familiar faces and words. They found that, when pre-experimentally familiar faces and words were repeated prior to being associated with source contexts, participants were less accurate learning item-source pairings. By contrast, item-source associations for novel/unfamiliar items were improved following repetition. They confirmed the role of familiarity by subsequently familiarizing participants with otherwise novel items prior to the repetition and source-learning phases. This familiarization procedure eliminated the benefit of repetition prior to source learning: Participants were better able to learn item-source pairings for truly unfamiliar items.

Although Lee et al. (2020) examined the effects of repetition prior to learning item-source pairings, the extant literature has largely focused on the effects of repetition on the item-source learning itself. Unsurprisingly, when items are repeatedly paired with their sources, people better learn the associations (Tulving & Schacter, 1990). Repetition may afford the opportunity for item-source pairs to become associated with more semantic and contextual details that aid later retrieval efforts (Poppenk & Norman, 2012). In contrast, pre-experimentally familiar items likely already have strong mental representations, such that more exposure does not enhance the likelihood of remembering them. Instead, prior familiarity might decrease source accuracy because participants may allocate fewer attentional resources when one or both members of item-source pairs are familiar (Ranganath & Rainer, 2003).

In complex source memories, such as memory for where and how you know a particular person, the face is not the only element that can provoke feelings of familiarity. For example, consider a college student who has several classes with other students in the same major. Although individual professors may be strongly associated with a single context (i.e., the class they teach), the other students in their cohort may be weakly associated with multiple contexts (i.e., different classrooms and, most likely, other locations on campus). Prior research consistently shows that stronger associations between items and sources increase source memory accuracy (e.g., Kelley & Wixted, 2001; Light, et al., 2004; Mitchell & Zaragoza, 1996). For example, Buchler et al. (2008) showed that repeating presentations of unrelated paired associates (e.g., *sky-chemical*) during encoding led to strengthened item memories (i.e., memory for *sky*) and more accurate memories for the original context pairing (e.g., *sky-chemical* vs. *sky-pencil*). The repetition-strength relationship was only observed when items and contexts were consistently paired together. When items appeared with multiple contexts (e.g., *sky-chemical, sky-waffle, sky-toothbrush*), associative memory was decreased (e.g., a fan effect, Anderson, 1974). The fact that highly familiar items with weak associative memory led to more source errors underscores the importance of both item and associative memory for correct source judgments. To date, no work has examined how the type of familiarity (i.e., experimental vs. pre-experimental) and associative memory strength impact source memory accuracy for faces.

## Current Studies

The goal of the current studies was to examine how pre-experimental versus experimental familiarity influence recognition and source memory. Experiment 1 compared how familiarity type (pre-experimental or experimental) and repetition influence recognition accuracy, using known and unknown faces to manipulate familiarity. To avoid testing picture memory, which is notoriously accurate (Brady et al., 2008; Kuhbandner et al., 2017; Standing, 1973), all faces that were studied were depicted by different photographs at test. To preview the results, we found that participants were better able to recognize pre-experimentally familiar faces compared to experimentally familiar faces, but that repetition increased accuracy for both familiarity types. Experiment 2 expanded on these findings by pairing pre-experimentally familiar and unfamiliar faces with location backgrounds. We manipulated the associative strength between face-location pairings by repeating faces with consistent (i.e., strong associations) or different (i.e., weak associations) locations. Experiment 2 revealed that pre-experimentally familiar faces were associated with higher false alarm rates when associative strength was weak or when the face-location pairing was only seen once relative to faces made familiar through intraexperimental repetition.

## Experiment 1

### Method

#### Design

Experiment 1 conformed to a 2 (Familiarity Type: Pre-Experimental, Experimental) × 3 (Repetition: thrice, once, new) within subjects design. The dependent variable of interest was participants’ old/new judgment accuracy.

#### Participants

An a priori power analysis was conducted using MorePower (Campbell & Thompson, 2012). Using an α of .05, a β of .8, and looking for a medium effect size, the recommended sample size was 52 participants. A sample of 76 participants were recruited from a large, southern university. Participants were recruited through the university’s Sona-Systems platform and were given course credit in exchange for completing the study. Participants’ *M*_age_ was 20.59 years (*SD* = 2.01). Most (76.3%) participants self-identified as female with the remaining identifying as male. Overall, 35.5% of participants identified as Hispanic, 32.9% identified as Caucasian, 27.6% identified as Black, with the remaining identifying as “Other.”

#### Face Stimuli

Forty famous American faces and 40 famous faces of European actors were selected for use in the study based on an iterative selection process. To acquire the famous American faces, five college-aged research assistants created a list containing the names of as many famous people as they could, aiming to identify celebrities who should be easily recognizable and nameable by other college students. Research assistants also created a second list containing names of famous foreign actors that they identified by Google searching European television stars. This list was used to create what we will refer to as the “unfamiliar” faces. We used famous non-American actors to avoid confounds related to celebrity status and attractiveness or memorability. Combined, the initial lists contained 415 famous American and famous European names. From this list, 10 new research assistants rated whether they (1) knew and could name the face or a character the actor played, (2) knew the face but could not name them, or (3) have never seen the face before. This approach is commonly utilized in studies examining memory for pre-experimentally familiar faces (e.g., Lee et al., 2020). Famous American faces were included in the next round of norming if the face was rated as being easily recognizable by at least 7 raters. European famous faces were included if they were rated as not having been seen before by at least 7 raters. These criteria produced 124 faces for the next round of photograph norming.

The 10 research assistants who rated the faces next found two photographs for each of the 124 selected celebrities. We used two pictures of each person, one at study and one at test, to ensure that participants were relying on face recognition, rather than photograph recognition (assignment of photographs to study or test was counterbalanced across participants during the actual experiment). Selection criteria for photographs included pose (head-on photos), roughly around the same age (i.e., no large time gaps between the pictures), lack of distinctive jewelry, facial expressions, etc., and good quality (i.e., not pixelated). All backgrounds were removed, and the photographs were cropped at the shoulders such that only the neck and head of each celebrity were shown to participants. Both pictures of each celebrity were presented in a Qualtrics survey to a sample of 34 psychology students who completed it in exchange for course credit. Participants in the norming sample saw both pictures of each celebrity and were asked (1) if they have seen the person before and (2) if they answered yes, to name the person if possible. Famous American faces were included in the final stimulus set if 85%–100% of participants could both identify them and name them; we selected the 40 most known faces and these became the “familiar” faces used in the current studies. Famous European faces were only included if fewer than 10% of participants indicated that they knew the face and could provide a name; we selected the 40 least known faces, which became the unfamiliar faces. For both the familiar and unfamiliar picture sets, faces were randomly assigned to be seen once, thrice, or not at all during the study phase. This resulted in six different item types: (1) familiar faces that were seen thrice, (2) familiar faces that were seen once, (3) familiar faces that were not seen at all, (4) unfamiliar faces that were seen thrice, (5) unfamiliar faces that were seen once, and (6) unfamiliar faces that were not seen at all.

During the study phase, participants saw a series of 80 faces. Although participants saw 80 faces, they only saw 40 identities (20 famous American, half of which were shown three times, and 20 famous European, half of which were shown three times). At test, participants provided 40 old/new judgments for all previously seen identities and 40 old/new judgments for the unseen identities (20 famous American and 20 famous European).

#### Procedure

After providing informed consent, participants read instructions telling them that they would study a series of faces for purposes of a later recognition test. Once participants indicated they were ready to continue, they were shown a series of 80 faces, one at a time, for 2 s each. After the 80 study trials, participants engaged in a 3-min distractor task where they solved math problems before moving on to the test phase.

During the test phase, participants were instructed that they would see a series of faces and that their task was to indicate whether they saw that person during the study portion. Importantly, participants were told that their decisions should be based on identities, rather than pictures, because no photographs would repeat across the study and test phases of the experiment. Participants provided their judgments on a six-point Likert scale with anchors at 0 (= *completely sure new*) and 100 (= *completely sure old*). After indicating they understood the instructions, participants provided their old/new judgments for 80 faces (half old). Upon completion of the memory test, participants provided demographic information and then were thanked and debriefed.

### Results and Discussion

#### Hits and False Alarms

To examine how pre-experimental and experimentally-induced familiarity affect memory for faces, a 2 (Familiarity Type: Pre-Experimental, Experimental) × 2 (Repetition: thrice, once) within-subjects ANOVA was conducted on participants’ hit rates, and a paired sample *t*-test was conducted on participants’ false alarm rates to new faces. To calculate hit and false alarm rates, participants’ responses to the six-point Likert scale were recoded such that responses of 0, 20, and 40 were coded as “new” (represented by a dummy code of 0) and responses 60, 80, and 100 were coded as “old” (represented as 1). The number of “old” responses for each of the three item types was summed and divided by the total number of items seen to create a proportion for each item type (e.g., a participant responding “old” to six of the 10 pre-experimentally familiar faces would have a hit rate of .6).^1^ This was done for all six of the item types, resulting in six scores per participant.

For all analyses reported in this paper, alpha was set at .05 and follow-up Bonferroni comparisons were used where appropriate. Any violations of sphericity were addressed by Greenhouse-Geisser corrections where applicable. Bayes Factors values for all analyses were calculated via https://tomfaulkenberry.shinyapps.io/psystat/ (Faulkenberry, 2021, 2022; Faulkenberry & Brennan, 2023). Bayes Factors values should be interpreted consistently with Jeffreys (1961) such that values of 1–3 represent anecdotal evidence in favor of the hypothesis, 3–10 are representative of moderate support, 10–30 as strong support, 30–100 as very strong support, and over 100 as decisive evidence in support of the hypothesis. The data for both experiments can be found at: https://osf.io/fc9p2/?view_only=922c18d7b1784ea7994abbe9eb7567b5.

The *M* and *SE* for all item types can be seen in Table 1.

**Table 1:** *M* and *SE*s (in brackets) for participants’ hit and false alarm rates in Experiment 1

Repetition	Familiarity type	
	Pre-experimental	Experimental
Thrice	0.91 (0.02)	0.51 (0.03)
Once	0.79 (0.02)	0.30 (0.02)
New	0.11 (0.02)	0.17 (0.02)

The ANOVA on hit rates revealed significant main effects of both Familiarity Type, *F*(1, 75) = 405.82, *p* < .001, η^2^ = .84, BF_10_ > 100, and Repetition, *F*(1, 75) = 98.36, *p* < .001, η^2^ = .57, BF_10_ > 100, as a well as a significant interaction between the two, *F*(1, 75) = 11.91, *p* = .005, η^2^ = .10, BF_10_ = 31.50. Participants were more likely to correctly recognize pre-experimentally familiar faces (*M* = 0.85, *SE* = 0.02) than experimentally familiar faces (*M* = 0.40, *SE* = 0.02). They were also more accurate for faces shown thrice (*M* = 0.71, *SE* = 0.02) as opposed to only once (*M* = 0.55, *SE* = 0.02).

Follow-up *t*-tests separated by Item Type were conducted to evaluate the significant interaction. For faces shown once, participants were better at recognizing pre-experimentally (*M* = 0.79, *SE* = 0.02) faces than experimentally familiar faces (*M* = 0.30, *SE* = 0.02), *t*(75) = 17.21, *p* < .001, *d* = 2.51. Similarly, for faces shown three times, memory was better for pre-experimentally (*M* = 0.91, *SE* = 0.02) than experimentally familiar faces (*M* = 0.51, *SE* = 0.02), *t*(75) = 15.73, *p* < .001, *d* = 2.21.

Finally, the paired samples *t*-test on participants’ false alarms to new faces revealed that participants were more likely to falsely recognize novel unfamiliar (*M* = 0.17, *SE* = 0.02), rather than pre-experimentally familiar, faces (*M* = 0.11, *SE* = 0.02), *t*(75) = 3.39, *p* = .001, *d* = .39, BF_10_ = 22.30.

#### Signal Detection Analyses

In addition to the analyses for participants’ hits and false alarms, we also calculated estimates of participants’ discriminability (*d*′) and response bias (*c*). To create these estimates, we used participants’ hit rates from each of the four old item types and used the corresponding new items as the false alarm rate (e.g., the pre-experimental thrice and once faces used the pre-experimentally familiar new faces as their false alarm rates). Higher values of *d*′ are indicative of better discriminability. Participants with more positive values of *c* were more conservative in their responding. Separate 2 (Familiarity Type: Pre-Experimental, Experimental) × 2 (Repetition: thrice, once) within-subjects ANOVAs were conducted on participants’ *d′* and *c* scores.

#### Discriminability

The ANOVA on participants’ *d*′ scores revealed main effects of both Familiarity Type, *F*(1, 75) = 374.53, *p* < .001, η^2^ = .83, BF_10_ > 100, and Repetition, *F*(1, 75) = 87.99, *p* < .001, η^2^ = .54, BF_10_ > 100. The interaction was not significant, *p* = .90, BF_01_ = 6.79. Participants had better discriminability for pre-experimentally (*M* = 2.79, *SE* = 0.12) compared to experimentally (*M* = 0.80, *SE* = 0.07) familiar faces. Discriminability was also higher when faces were seen thrice (*M* = 2.11, *SE* = 0.09), as opposed to once (*M* = 1.48, *SE* = 0.09).

#### Response Bias

Similar to the discriminability analyses, there were main effects of Familiarity Type, *F*(1, 75) = 94.19, *p* < .001, η^2^ = .56, BF_10_ > 100, and Repetition, *F*(1, 75) = 87.97, *p* < .001, η^2^ = .54, BF_10_ > 100, on participants’ response bias. The interaction, again, was not significant, *p* = .90, BF_01_ = 6.79. Participants responded more conservatively when responding to experimentally (*M* = 0.75, *SE* = 0.07) rather than pre-experimentally (*M* = 0.08, *SE* = 0.05) familiar faces. Participants were also more conservative in their responses for faces that had been seen once (*M* = 0.57, *SE* = 0.05) rather than thrice (*M* = 0.26, *SE* = 0.05).

The data from Experiment 1 are consistent with prior work (e.g., Bird et al., 2011; Bruce, 1982; Dobbins & Kroll, 2005; Ellis et al., 1979; Klatzky & Forrest, 1984; Schwaninger et al., 2002), showing higher accuracy for pre-experimentally familiar faces compared to faces that were made familiar through repeated exposures. Although repetition increased correct recognition rates for both pre-experimentally and experimentally familiar faces, previously unfamiliar faces benefitted more: Relative to known individuals, whose recognition rates increased by .12 with repetition, recognition for previously unknown faces increased by .21. Despite this, recognition of experimentally familiarized, but previously unknown, faces never approached the same hit rates as faces that were previously familiar, highlighting a limitation in experimental techniques designed to induce familiarity. These data, however, only speak to isolated item memories. The effects of different types of familiarity and familiarization techniques for source memory are explored in Experiment 2.

## Experiment 2

Experiment 2 expands on Experiment 1 by pairing faces with various locations to examine the effects of familiarity on source memory. We also manipulated the strength of association between face-location pairings by having some faces shown three times in the same location (consistent), three times but in three different locations (variable), or only once (single). Although these experimental labels may seem contrived, they map onto real-world interactions. Returning to the college student example, faces seen three times in a consistent location are akin to a student seeing their professor in the same classroom throughout the semester. By contrast, faces seen multiple times in variable locations are akin to the student seeing unfamiliar members of their cohort in other classes and around campus. To capture this variability, participants in Experiment 2 could see old faces paired with old locations, old faces paired with new locations (we call these pairs “rearranged” in the Methods), and entirely new faces. In this way, Experiment 2 provides insight into how source memory might differ as a function of familiarity type and the strength of the association between the face and location.

### Method

#### Design and Participants

The design for Experiment 2 conformed to a 2 (Familiarity Type) × 7 (Repetition: old-consistent, old-variable, old-single, rearranged-consistent, rearranged-variable, rearranged-single, new) within-subjects design, with the dependent variables being participants’ old/new accuracy.

Although the design conformed to a 2 (Familiarity Type) × 7 (Repetition), the analyses of interest were a 2 (Familiarity Type: Pre-Experimental, Experimental) × 3 (Old Pairing Repetition: old-consistent, old-variable, old-single) conducted on participants’ hit rates and a 2 (Familiarity Type) × 4 (Prior Pairing Repetition: rearranged-consistent, rearranged-variable, rearranged-single, new) conducted on participants’ false alarm rates. Because of this, power analyses were conducted to account for the comparison that would require the largest number of participants. Using an α of .05, a β of .8, and looking for a medium effect size, the suggested sample size was 52 participants. Because of the decrease in the number of stimuli populating each item type, we permitted oversampling by collecting data until the end of the semester. As such, 100 participants completed Experiment 2 in exchange for course credit. Participants were recruited through the university’s Sona-Systems platform. *M*_age_ was 20.61 years (*SD* = 3.23). Again, most (77.2%) participants self-identified as female with the remaining identified as male. Overall, 39% of participants identified as Caucasian, 33% identified as Hispanic, and 24% identified as Black, with the rest identifying as “Other.”

#### Face-Location Pairs

**Figure 1:**
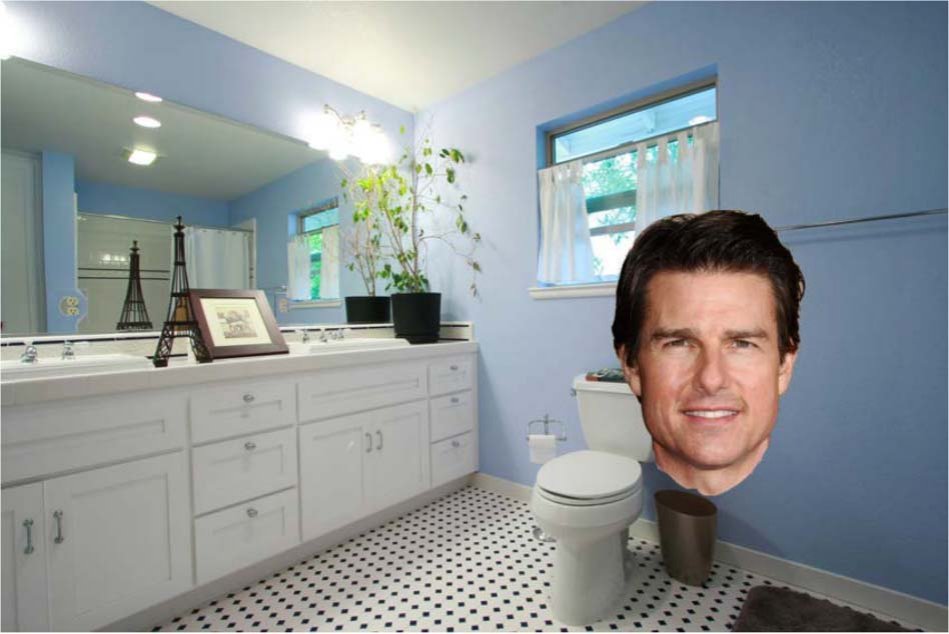
Example of face-background pairing seen by participants in Experiment 2.

A sample of 96 location pictures were obtained for Experiment 2. These pictures were obtained from Google searching various locations (e.g., bathroom, beach) and edited to conform to a standard size of 1,010 × 673 pixels. Locations were selected such that each location was semantically distinctive, easily recognized, and nameable by a group of eight research assistants. The faces from Experiment 1 were superimposed on top of locations to create face-location pairs (see Figure 1 for an example).

We again manipulated how many times the faces were seen at study (except for truly new items, which were not shown at study). Some faces were shown three times with the same background (hereby referred to as “consistent” items). Other faces were seen three times during study, but with three different backgrounds (e.g., Face A may have appeared with the beach, the bathroom, and the jail; hereby referred to as “variable” items). Finally, some faces were shown only once in a single location at study (hereby referred to as “single” items).

The inclusion of locations resulted in several item type combinations during the memory test (see Table 2). Old items were defined as test faces that appeared with one of their studied locations (i.e., old-consistent, old-variable, old-single). Rearranged items were defined as faces previously paired with a different location (i.e., rearranged-consistent, rearranged-variable, rearranged-single). New items were defined as faces that were previously unseen. This resulted in seven different face-location item types. There were seven counterbalanced conditions so that each face could act as each of the seven different item types. For each item type, participants saw four face-location pairs.

**Table 2:** Examples of study and test items for the Experiment 2 item strength association manipulation

Item type	Study presentation	Old test	Rearranged test
Consistent	Face A - Beach Face A - Beach Face A - Beach	Face A - Beach	Face A - Helicopter
Variable	Face B - Circus Face B - Sky Face B - Igloo	Face B - Circus	Face B - Meadow
Single	Face C - Pool	Face C - Pool	Face C - Jail
New	Absent	—	Face D - Bathroom
*Note*. Participants only saw one version of the old test items, an old pairing or a rearranged pairing, never both.			

#### Procedure

The procedure for Experiment 2 was similar to Experiment 1, except that faces at both study and test were paired with location backgrounds. Participants saw 64 sequential face-location pairings during the study phase presented in random order for 2 s each. They engaged in a 3-min distractor task before moving to the testing phase.

Participants read similar test instructions but were now told that their task was to indicate whether the person was shown in the same location that they were seen with during study. If participants believed that the identity had been seen with the currently-shown location during study, they indicated that the item was old. If they believed that either the identity or location had been seen with another item at study, they were to indicate that the item was new. Responses were again provided on a 6-point Likert scale. Participants provided 56 old/new judgments before providing demographic information and being thanked and debriefed.

### Results and Discussion

Repeated measures ANOVAs were conducted on participants’ hit and false alarm rates, discriminability, and response bias scores, all of which were calculated as in Experiment 1. The analysis for participants’ hit rates conformed to a 2 (Familiarity Type: Pre-Experimental, Experimental) × 3 (Old Pairing Repetition: old-consistent, old-variable, old-single) within-subjects ANOVA. The analysis for participants’ false alarms conformed to a 2 (Familiarity Type) × 4 (Prior Pairing Repetition: rearranged-consistent, rearranged-variable, rearranged-single, new) within-subjects ANOVA. The signal detection analyses were conducted using a 2 (Familiarity Type: Pre-Experimental, Experimental) × 3 (Pairing Repetition: Consistent, Variable, Single) within-subjects ANOVA. Means and standard errors for Experiment 2 can be seen in Table 3.

**Table 3:** *M* and *SE* (in brackets) for participants’ hit and false alarm rates in Experiment 2

Repetition	Pre-experimental	Experimental
Old		
Consistent	0.89 (0.02)	0.57 (0.03)
Variable	0.63 (0.03)	0.34 (0.03)
Single	0.66 (0.03)	0.28 (0.03)
Rearranged		
Consistent	0.17 (0.02)	0.13 (0.02)
Variable	0.28 (0.03)	0.16 (0.02)
Single	0.15 (0.02)	0.08 (0.02)
New	0.04 (0.01)	0.03 (0.01)

The omnibus ANOVA on participants’ hit rates revealed significant main effects of both Repetition, *F*(2, 198) = 83.92, *p* < .001, η^2^ = .46, BF_10_ > 100, and Familiarity Type, *F*(1, 99) = 245.88, *p* < .001, η^2^ = .71, BF_10_ > 100. The interaction term was not significant, *p* = .10, BF_01_ = 10.54. Pairwise comparisons for the main effect of Repetition revealed that participants were more accurate at recognizing faces they had seen three times in the same location (old-consistent; *M* = 0.73, *SE* = 0.02) compared to faces seen three times in three different locations (old-variable; *M* = 0.48, *SE* = 0.02) or faces only seen once (old-single; *M* = 0.47, *SE* = 0.02), *p*s < .001. The difference between the old-variable and old-single items was not reliable, *p* = 1.00. The main effect of Familiarity Type revealed that participants were more accurate in recognizing pre-experimentally (*M* = 0.73, *SE* = 0.02) compared to experimentally familiar faces (*M* = 0.39, *SE* = 0.02), *p* < .001.

The complementary ANOVA on participants’ false alarm rates revealed main effects of both Prior Repetition, *F*(2.60, 256.99) = 39.66, *p* < .001, η^2^ = .29, BF_10_ > 100, and Familiarity Type, *F*(1, 99) = 27.97, *p* < .001, η^2^ = .22, BF_10_ > 100, as well as a significant interaction, *F*(2.60, 257.29) = 4.73, *p* = .005, η^2^ = .05, BF_10_ = 1.22. The main effect of Prior Repetition revealed that participants were more likely to false alarm to faces that were seen in three different locations but shown in a fourth location at test (rearranged-variable; *M* = 0.22, *SE* = 0.02) compared to the other three item types, *p*s < .01. In contrast, completely novel faces (*M* = 0.04, *SE* = 0.01) were associated with lower false alarm rates compared to the rearranged-consistent (*M* = 0.15, *SE* = 0.02) or rearranged single items (*M* = 0.12, *SE* = 0.01), *p*s < .001. The comparison between the latter item types was not significant, *p* = .19. Although Experiment 1 revealed lower false alarm rates in item memory tests for pre-experimentally familiar faces, the main effect of Familiarity Type on associative false alarms in Experiment 2 revealed that participants were more likely to false alarm to pre-experimentally familiar (*M* = 0.16, *SE* = 0.01) rather than experimentally familiar faces (*M* = 0.10, *SE* = 0.01). Although this result may suggest that participants are less able to associate previously familiar faces to contexts, the hit rate data suggest that the more likely source of the increased error rate lies in the familiarity itself: When faces are pre-experimentally familiar, the strong familiarity signal likely overrides the more laborious associative judgment.

**Figure 2:**
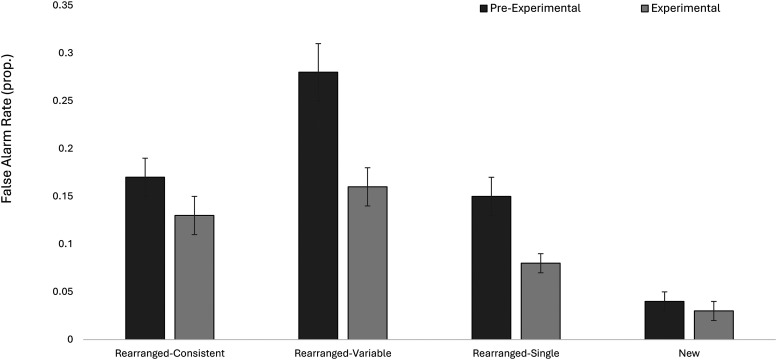
Means for the false alarm interaction data. Error bars denote the standard error of the mean.

To follow-up the significant interaction, a series of paired samples *t*-tests were conducted on participants’ false alarm rates for pre-experimentally and experimentally familiar faces. As shown in Figure 2, there were no familiarity-driven differences in false alarm rates for either the rearranged-consistent or the novel faces, *p*s > .10, BFs_01_ < 10. For the rearranged-variable items, participants were more likely to false alarm when the face was pre-experimentally (*M* = 0.28, *SE* = 0.03) compared to experimentally familiar (*M* = 0.16, *SE* = 0.02), *t*(99) = 4.51, *p* < .001, *d* = .50, BF_10_ > 100. A similar pattern was observed for the rearranged-single items such that participants provided more false alarms when the face was pre-experimentally familiar (*M* = 0.15, *SE* = 0.02) relative to the experimentally familiar faces (*M* = 0.08, *SE* = 0.01), *t*(99) = 3.45, *p* = .001, *d* = .41, BF_10_ = 26.46. These results show that, when participants are familiar with a face, but the association between that face and the context from which it is known is weak, they are more likely to falsely recognize that face as being associated with a new context.

#### Signal Detection Analyses

Similar to Experiment 1, repeated measures ANOVAs were conducted on participants’ *d’* and *c* scores. Correct “old” responses to faces that had been shown in the same location at study and test (i.e., “old” items) served as the hit rates while incorrect “old” responses to the corresponding “rearranged” pairs served as the false alarms (e.g., the old pre-experimental consistent items acted as the hits and the rearranged pre-experimental consistent items were the false alarms), resulting in a total of 6 *d*′ and *c* scores per participant that were used in each analysis.

#### Discriminability

Mirroring Experiment 1, there were main effects of both Familiarity Type, *F*(1, 99) = 105.63, *p* < .001, η^2^ = .52, BF_10_ > 100, and Pairing Repetition, *F*(2, 198) = 59.10, *p* < .001, η^2^ = .37, BF_10_ > 100. The interaction was not significant, *p* = .08, BF_01_ = 7.72. Similar to Experiment 1, discriminability was better for pre-experimentally (*M* = 1.57, *SE* = 0.08) rather than experimentally (*M* = 0.82, *SE* = 0.06) familiar faces. Discriminability was highest when faces were shown in the same location three times than either of the other pairing repetition types, *p*s < .001. Faces that were only shown once in a single location (*M* = 1.07, *SE* = 0.08) also had better discriminability than faces seen in three different locations (*M* = 0.79, *SE* = 0.08).

#### Response Bias

Analyses on participants’ response bias revealed the main effects of Familiarity Type, *F*(1, 99) = 208.68, *p* < .001, η^2^ = .68, BF_10_ > 100, and Pairing Repetition, *F*(1.88, 185.72) = 51.64, *p* < .001, η^2^ = .34, BF_10_ > 100. The interaction was not significant, *p* = .23, BF_01_ = 23.12. In line with Experiment 1, participants were more conservative in their responses when responding to the experimentally (*M* = 0.73, *SE* = 0.04) familiar faces rather than the pre-experimentally (*M* = 0.11, *SE* = 0.04). Participants were most conservative for faces seen only once (*M* = 0.62, *SE* = 0.04) compared to the other item types, *p*s < .001. Similarly, responses for variable (*M* = 0.45, *SE* = 0.04) faces were more conservative than those for consistent faces (*M* = 0.18, *SE* = 0.04).

## General Discussion

The current studies examined how pre-experimental and experimental familiarity influence face recognition and source memory. Experiment 1 revealed that participants were better able to recognize faces that were pre-experimentally familiar, relative to those that were pre-experimentally unfamiliar, but made familiar through the experiment context. In Experiment 2, we tested how familiarity affects source memory. Specifically, we sought to explore the roles of both familiarity and association strength on participants’ ability to discern whether test pairings were presented together earlier. To that end, we paired familiar and unfamiliar faces with pictures of locations, such that some faces appeared with multiple consistent locations (strong associations) and others appeared with multiple varied locations (weak associations). Although participants had higher hit rates for pre-experimentally familiar faces, they were also more likely to false alarm when those faces had weak associations with their source context. These data reveal important nuances in how familiarity affects face and source memory.

The data from Experiment 1 replicate earlier work showing people are better at recognizing faces that have some pre-experimental familiarity (e.g., Bird et al., 2011; Klatzky & Forrest, 1984). Although repeatedly presenting pre-experimentally unfamiliar faces increased participants’ recognition ability, their accuracy was still not as high as when they studied pre-experimentally familiar faces only once. Many factors have been implicated in the benefits of familiarity for face recognition. For example, familiar individuals may be associated with enhanced conceptual knowledge that benefits encoding processes (Schwartz & Yovel, 2019). Prior work has often treated familiarity as a binary factor, such that faces either are or are not familiar. The current study adds to the growing literature investigating the more nuanced nature of real-world familiarity (e.g., Pica et al., 2018; Vallano, Pettalia, et al., 2019; Vallano, Slapinski, et al., 2019). By presenting pre-experimentally and experimentally familiar faces once or thrice, we were able to examine the effects of repetition (i.e., increasing familiarity) for both known and unknown faces. This approach helps to create a continuum of familiarity such that faces that are unknown to participants will also vary in their degree of familiarity. Although the finding that increased repetition of previously unfamiliar faces improved accuracy is not surprising, the fact that accuracy also increased for the pre-experimentally familiar faces shows that even faces with stronger mental representations benefit from repeated exposure. Prior familiarity also protected faces from false recognition: Relative to unfamiliar faces, pre-experimentally familiar faces were less likely to be mistakenly recognized if they were not shown during the study phase. Thus, these data replicate and extend prior research examining types and degrees of familiarity on face recognition.

The ability to discriminate between old and novel faces is an important task, but people must also often remember the context in which the person was encountered. Experiment 2 examined source memory for both familiar and previously unfamiliar faces that had been paired with various locations. Similar to Experiment 1, pre-experimentally familiar faces were associated with higher hit rates. The protective benefits of familiarity, however, did not emerge. Instead, previously familiar faces were associated with *higher* associative false alarm rates than faces made familiar only through the experimental context. Associative strength influenced accuracy such that strongly associated face-location pairs (i.e., faces shown three times with the same location) resulted in more hits and fewer false alarms. In contrast, faces that were seen multiple times, but with different locations each time, were the most likely to result in mistaken association judgments. This was particularly true for the pre-experimentally familiar faces. This finding is consistent with prior work showing that weaker memorial associations result in more source confusion (Buchler et al., 2008). In addition to the weak associative strength between the faces and locations, it is possible that pre-experimentally familiar faces did not receive the same amount of attentional allocation during the encoding (Ranganath & Rainer, 2003). For example, if pre-experimentally familiar faces feel inherently more memorable, participants may have been less motivated to commit those face-location pairs to memory, falsely believing that they would remember later (e.g., as in a distinctiveness heuristic).

While the data from Experiment 1 replicate prior work examining memory for familiar and unfamiliar faces, the data from Experiment 2 build upon findings from Lee et al. (2020), who found lower source learning for pre-experimentally familiar faces that were repeated prior to being associated with a source context. Across two experiments, they found that repetition-induced familiarity only benefited subsequent source learning for unfamiliar items. Our data add to these findings by showing the damaging effects of repetition for familiar items *during* learning. Specifically, we found that repeating familiar faces with multiple contexts impaired associative learning and produced higher rates of false recognition.

The current studies may also provide insight into how memory for familiar and unfamiliar faces may operate in more applied settings such as in eyewitness recognition. Specifically, these studies highlight two complex issues with eyewitness memory: the need to recognize a previously seen face and to be able to associate the face with the correct source. Although abundant research has explored the circumstances in which unfamiliar innocent people are accused of crimes, Experiment 2 was a closer experimental analogue to when an innocent *familiar* person is accused of a crime. Instances in which witnesses mistakenly identify familiar, but innocent individuals are known as unconscious transference errors (e.g., Carlson et al., 2023; Read et al., 1990; Ross et al., 1994; Wulff & Hyman, 2022). Although it may seem that unconscious transference errors only occur for familiar, but unknown (i.e., not nameable) individuals, case studies demonstrate that these errors can also occur for familiar and identifiable individuals. For example, Dewey and Gerald Davis were erroneously accused, and subsequently convicted, of abduction, sexual abuse, and sexual assault by a family friend, despite them knowing both Dewey and Gerald’s names and why they were familiar to them. Experiment 2 provides insight into how these errors might occur.

In the present study, unconscious transference occurred when participants falsely claimed that familiar faces had been paired with new locations, and this effect was exaggerated when the faces were pre-experimentally familiar. These errors could not arise because the participants did not remember seeing the face, as they would have then labeled the pair as “new.” They could also not emerge from the participants blending identities two people, as the new locations paired with faces at test were not previously shown with any other faces. Instead, similar to other work (Carlson et al., 2023), these data suggest that these errors are due to poor source memory. Specifically, unconscious transference errors that act upon familiar individuals are most likely due to weak associative binding between the familiar individual and the source or sources from which they are known.

Although the notion that it should be easy to remember contextual information associated with known individuals seems intuitive, the data from the current studies suggest that familiar individuals are not immune to source errors. While familiar faces are easily recognizable, the current studies show that pre-experimentally familiar faces are at higher risk of mistaken source attributions when they are weakly associated with a specific context. For example, a college student with multiple classes with the same cohort of other students should be more likely to falsely accuse another student of a misdeed than the professor they associate strongly with only one context. Although this example is contrived, real individuals have been accused of crimes simply for being familiar but not strongly associated with the context that made them familiar. The current studies represent one of the first steps toward better understanding these errors and the different ways that both pre-experimental and experimental familiarity impact both item and source memory.

## Footnotes

^1^
We intended to run analyses for participants’ confidence as well, but there were not enough responses per item types to reliably calculate these analyses.

## Notes

**Publication Ethics:** Informed consent was obtained from all participants included in the study.

All procedures in studies involving human participants were performed in accordance with the ethical standards of the SHSU’s Human Research Ethics Committee.

**Authorship:** Daniella Cash, conceptualization, data curation, formal analysis, investigation, methodology, project administration, resources, software, supervision, validation, visualization, writing- original draft, writing – review and editing; Megan Papesh, conceptualization, investigation, methodology, validation, visualization, writing- original draft, writing – review and editing; Alan Harrison, data curation, resources, software, writing – review and editing. All authors approved the final version of the article.

**Open Science:** The data for both experiments can be found at: https://osf.io/fc9p2/?view_only=4356d5d37f844d02979e3fef9fb19866 (Cash, 2024).

My manuscript contains no experiment with a completely executed preregistration.
